# Influence of *FOXP3* rs2280883 and rs3761548 Variants on IL-10 and TGF-β1 Serum Levels and Plaque Psoriasis Risk in the Mexican Population

**DOI:** 10.3390/ijms26051789

**Published:** 2025-02-20

**Authors:** Jorge Hernández-Bello, Miriam Sarahi Preciado-Aguiar, José Francisco Muñoz-Valle, Christian Johana Baños-Hernández, Samuel García-Arellano, Anabell Alvarado-Navarro

**Affiliations:** 1 Instituto de Investigación en Ciencias Biomédicas, Centro Universitario de Ciencias de la Salud, Universidad de Guadalajara, Guadalajara 44340, Jalisco, Mexico; jorge.hernandezbello@cucs.udg.mx (J.H.-B.); drjosefranciscomv@cucs.udg.mx (J.F.M.-V.); johana.banos@academicos.udg.mx (C.J.B.-H.); samuel.g.arellano@gmail.com (S.G.-A.); 2 Centro de Investigación en Inmunología y Dermatología, Centro Universitario de Ciencias de la Salud, Universidad de Guadalajara, Guadalajara 44340, Jalisco, Mexico; miriam_spa93@hotmail.com

**Keywords:** plaque psoriasis, *FOXP3* variants, Tregs, IL-10, TGF-β1

## Abstract

Plaque psoriasis (PP) is a chronic immune-mediated skin disorder with a genetic basis, characterized by abnormal T-cell responses. This study investigated the role of *FOXP3* gene variants rs2280883 and rs3761548 in T-cell regulation through their effects on IL-10 and TGF-β1 cytokine levels and their association with PP risk. A case-control study was conducted, including 101 individuals with PP and 106 healthy controls from the Mexican population. Genotyping of *FOXP3* variants was performed using PCR-RFLP, and cytokine levels were measured with ELISA kits. Significant differences in allele and genotype frequencies of the rs2280883 variant were observed between PP patients and controls, suggesting an association with an increased risk of PP. IL-10 levels were found to be elevated in PP patients, regardless of *FOXP3* gene variants, indicating that cytokine dysregulation in PP may involve alternative pathways independent of FOXP3-mediated regulatory T-cell (Treg) function. No significant differences were detected in TGF-β1 levels or rs3761548 genotype frequencies across the study groups. In conclusion, the rs2280883 variant in the *FOXP3* gene is significantly associated with a higher risk of developing PP in the Mexican population, while dysregulated IL-10 levels suggest a complex cytokine interaction beyond Treg activity.

## 1. Introduction

PP is a chronic immune-mediated skin disorder characterized by hyperproliferative lesions and extensive inflammation [1]. It affects approximately 2–3% of the global population, with an estimated 125 million individuals affected worldwide. The global prevalence of PP is increasing across various racial, ethnic, and geographical groups, with White individuals being nearly twice as likely to develop psoriasis compared to non-White individuals. In the United States, the prevalence among White populations is reported at 3.6%, whereas in non-White populations, it ranges from 1.5% to 3.1% [2,3,4,5]. However, epidemiological data remain limited outside the U.S., particularly in Latin America and the Caribbean [2,3].

The economic impact of psoriasis is substantial, with indirect costs, such as lost productivity, often exceeding direct healthcare expenses. In Colombia, annual indirect costs per patient have been estimated at USD 8476, surpassing medical costs. Similarly, in Argentina, annual direct costs for moderate-to-severe cases have been reported at USD 5326. Although specific data for Mexico remain scarce, similar trends are expected [6]. Additionally, psoriasis significantly affects quality of life, as individuals with the condition are at a higher risk of developing depression, anxiety, and stress [7].

The etiology of psoriasis remains unclear; however, it is recognized as a complex disease driven by genetic predisposition and environmental triggers. Genetic studies have identified multiple susceptibility loci, with *PSORS1* genes, particularly *HLA-Cw6*, being most strongly associated with early-onset psoriasis. Genome-wide association studies (GWASs) have further linked psoriasis to inflammatory pathways, including NF-κB, TNF, and IL-23/TH17 [8]. Nonetheless, psoriasis typically manifests following exposure to triggering factors such as trauma [9], obesity [10], infections [11], stress [12], and medications [13]. The interaction between genetic susceptibility and environmental influences ultimately determines disease onset and severity. Fundamentally, psoriasis arises from a dysregulated immune response, with T cells playing a central role in its immunopathogenesis [8,14].

Regulatory T cells (Tregs), characterized by the expression of CD4, CD25, and the transcription factor FOXP3, play a central role in maintaining immune tolerance and preventing autoimmunity [15]. FOXP3 is predominantly expressed in Treg cells and functions as a “master regulator” of their polarization and immunosuppressive properties [16,17]. It is closely associated with regulatory cytokines such as IL-10 and TGF-β [18,19].

IL-10 and TGF-β1 play critical roles in suppressing inflammatory responses and promoting tissue repair [20], processes that are frequently disrupted in psoriatic lesions. Additionally, these cytokines contribute to the expansion of FOXP3^+^-induced Tregs, enhancing their CTLA-4 expression and suppressive capabilities to levels comparable to those of natural Tregs [21].

Several studies have highlighted the role of genetic variants within the *FOXP3* gene, including rs2280883 and rs3761548, in influencing *FOXP3* expression and function, thereby altering Treg activity and disrupting immune homeostasis in inflammatory and autoimmune diseases [22,23].

Research conducted in the Egyptian population has identified a significant association between the rs3761548 variant and psoriasis [24]. This association may be explained by the variant’s link to reduced IL-10 and TGF-β1 cytokine levels, which are essential for the Treg-mediated suppression of inflammation [25,26]. However, the associations between these genetic variants and disease susceptibility have shown inconsistencies or have been absent in different populations. This variation suggests that genetic influences on disease pathogenesis may differ significantly depending on genetic background, environmental factors, or both [22,23,27,28].

This study aimed to investigate the influence of the rs2280883 and rs3761548 variants in the *FOXP3* gene on IL-10 and TGF-β1 serum levels, as well as their association with the risk of developing plaque psoriasis in the Mexican population.

## 2. Results

### 2.1. Clinical and Demographic Characteristics of Study Groups

The clinical and demographic characteristics of the PP patients and controls are summarized in Table 1. The median age of the PP patients was 46 years (range: 35–57), with 51% identifying as male. The average disease duration was approximately 9 years, and the median Psoriasis Area and Severity Index (PASI) score was recorded at 10.9. Regarding treatment status at the start of the study, 67% of the PP patients were receiving treatment, while 33% were not undergoing any treatment. The control group had a median age of 45 years (range: 32–51), with a similar gender distribution to the PP group (*p* = 0.24).

### 2.2. Frequencies of the rs2280883 and rs3761548 FOXP3 Variants

The distribution of alleles and genotypes of *FOXP3* variants in PP patients and CSs is presented in Table 2. The GG genotype of the rs2280883 variant was the most frequent among PP patients (56%) and CSs (63%). Similarly, the CC genotype of the rs3761548 variant was the most prevalent in both groups (PP: 59% vs. CS: 58%). The Hardy–Weinberg equilibrium was maintained for both variants in the CS group (*p* > 0.05).

Significant differences in genotypic and allelic frequencies were observed for the rs2280883 variant. The AA genotype (OR 24.68, 95% CI 3.22–189.25, *p* < 0.0001) and A allele (OR 2.04, 95% CI 1.29–3.21, *p* = 0.001) were more prevalent among PP patients. A similar association was identified under a recessive inheritance model (OR 27.56, 95% CI 3.63–209.24, *p* < 0.0001).

### 2.3. IL-10 and TGF-β1 Serum Levels in PP Patients and CSs

**Figure 1:**
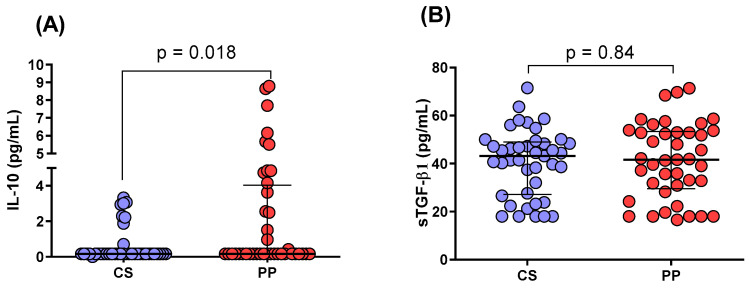
Comparison of IL-10 and TGF-β1 serum levels. (**A**) IL-10 serum levels in PP patients and CSs. (**B**) Comparison of TGF-β1 serum levels between PP patients and CSs. *p*-values were calculated using the Mann–Whitney U test. Data are presented as medians and interquartile ranges.

Significantly higher serum levels of IL-10 were observed in PP patients compared to CSs (Figure 1A, *p* = 0.018). However, no significant differences were detected in TGF-β1 serum levels between the two study groups (Figure 1B, *p* = 0.84). Additionally, analyses revealed no correlation between cytokine levels and PASI score, BMI, or treatment status among the participants.

### 2.4. Comparison of IL-10 and TGF-β1 Serum Levels by rs2280883 and rs3761548 FOXP3 Genotypes

**Figure 2:**
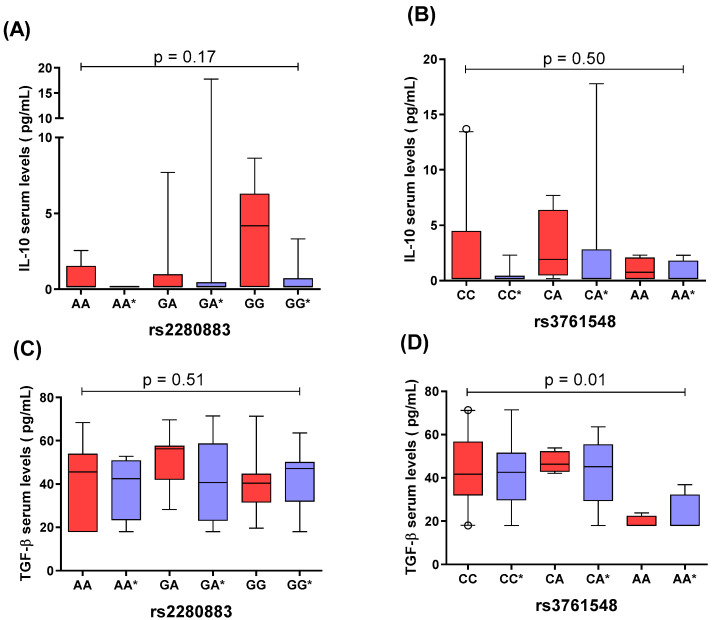
Distribution of serum cytokine levels across *FOXP3* gene genotypes. (**A**) IL-10 levels by rs2280883 genotype. (**B**) IL-10 levels by rs3761548 genotype. (**C**) TGF-β1 levels by rs2280883 genotype. (**D**) TGF-β1 levels by rs3761548 genotype. PP patients are represented by red-colored bars, while CSs (control subjects) are represented by purple-colored bars, with genotypes marked by an asterisk (*). Statistical analysis was conducted using the Kruskal–Wallis test, followed by Dunn’s multiple comparisons test to assess differences across groups. The box plots display data distribution from the fifth to the ninety-fifth percentile. The central box represents the median and quartiles, while the whiskers extend to show the range of data within these percentiles.

The comparative analysis of IL-10 and TGF-β1 serum levels, stratified by rs2280883 and rs3761548 genotypes in the *FOXP3* gene, revealed no statistically significant differences (Figure 2A–D). This lack of variation was observed both within each study group (intragroup) and between PP patients and CSs (intergroup). Notably, in Figure 2D, detectable differences emerged when all data groups were analyzed collectively. However, these differences did not persist in subsequent multiple comparison tests, indicating a lack of statistical significance in individual group comparisons.

A comparison of patients’ clinical characteristics based on genotype was conducted, and no significant differences were detected.

### 2.5. Linkage Disequilibrium Analysis

**Figure 3:**
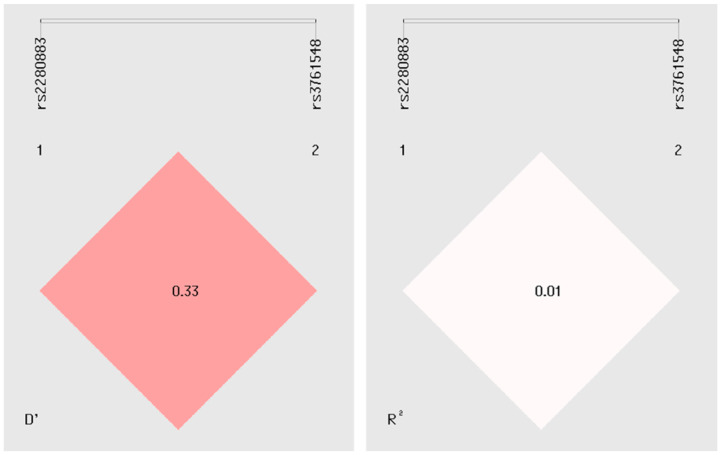
Linkage disequilibrium (LD) analysis of rs2280883 and rs3761548 *FOXP3* variants. The D’ and R^2^ values are displayed within the squares. A D’ or R^2^ value of 1 indicates complete LD between two markers, whereas a D’ value of 0 signifies complete linkage equilibrium.

The potential for haplotype construction was assessed by analyzing linkage disequilibrium (LD) between the *FOXP3* variants. The results yielded a D’ value of 0.3 and an R^2^ of 0.01, indicating negligible LD between the variants. These findings suggest that these genetic markers are inherited independently within the studied population, thereby limiting the applicability of haplotypic approaches for these specific variants in this context (Figure 3).

## 3. Discussion

The *FOXP3* gene variants, including rs2280883 and rs3761548, have been extensively studied in immune-mediated diseases such as systemic sclerosis [29], rheumatoid arthritis [30], Graves’ disease [31], and systemic lupus erythematosus [32]. The rs3761548 variant has also been linked to variations in *FOXP3* expression [33], which plays a pivotal role in Treg function and the maintenance of immune tolerance [16].

Regarding psoriasis, genetic evidence on the influence of *FOXP3* variants in disease susceptibility remains controversial, with studies reporting conflicting results. In an Egyptian population, the rs3761548 CC genotype and C allele were found to be significantly more frequent in psoriasis patients than in controls, suggesting a potential role in disease predisposition [24]. Conversely, a study conducted on Han Chinese patients reported an association between the rs3761548 AC and AA genotypes and an increased risk of psoriasis compared to the CC genotype. Furthermore, the rs2280883 GG genotype was identified as conferring an even higher risk (adjusted OR: 2.24) [27].

In contrast, studies conducted in other populations have yielded differing results. For instance, in a South Indian Tamil cohort, no significant association was observed between the rs3761548 variant and psoriasis risk [34]. These conflicting findings underscore the potential role of ethnogenetic variability in modulating the influence of *FOXP3* variants on psoriasis susceptibility.

In the present study, a significant association between the rs2280883 variant and an increased risk of developing PP was identified, whereas no such association was detected for the rs3761548 variant. Specifically, the rs2280883 AA genotype exhibited a strong association under both the codominant inheritance model (OR = 24.68; 95% CI: 3.22–189.25; *p* < 0.0001) and the recessive inheritance model (OR = 27.56; 95% CI: 3.63–209.24; *p* < 0.0001) when compared to the GG + AG genotypes. This significant association underscores the potential role of the rs2280883 AA genotype as a genetic marker for psoriasis risk in the Mexican population.

The variation in association patterns across populations underscores the complexity of *FOXP3*-mediated immune regulation in psoriasis. While studies on Egyptian and Han Chinese populations have suggested a strong role for *FOXP3* variants, the absence of an association in the South Indian Tamil cohort and the specific role of the rs2280883 AA genotype in the analyzed population highlight the importance of considering population-specific genetic and environmental factors. These findings reinforce the necessity of conducting more extensive and ethnically diverse studies to fully elucidate the genetic architecture of psoriasis and its underlying mechanisms.

LD analysis was performed to investigate the potential haplotypic effects of the rs2280883 and rs3761548 variants. No evidence of linkage was detected between these *FOXP3* variants, suggesting that their influence on psoriasis risk may operate independently. The absence of prior studies analyzing LD for these specific *FOXP3* variants limits the ability to compare these findings with existing literature. Nevertheless, the results provide a foundational reference for future research, emphasizing the potential independent contributions of these variants to genetic predisposition to psoriasis and other diseases.

Serum levels of IL-10 and TGF-β were measured alongside the analysis of *FOXP3* variants due to their close association with *FOXP3* [35,36] and their significant roles in the pathogenesis of psoriasis [37,38]. While both cytokines are key immunoregulatory molecules essential for maintaining immune tolerance, no significant differences in TGF-β levels were detected between PP patients and controls. However, IL-10 levels were found to be significantly higher (*p* = 0.018) in PP patients (median: 1.9 pg/mL) compared to controls (median: 0.6 pg/mL), potentially reflecting a compensatory anti-inflammatory response to chronic inflammation. This finding contrasts with previous studies reporting lower IL-10 levels in psoriasis patients [39,40]. The discrepancy may be attributed to differences in disease activity among study populations [41], the effects of prior or ongoing treatments that modulate cytokine responses [42], or the modest magnitude of IL-10 elevation observed in the analyzed cohort. Although IL-10 levels were elevated, they may still be insufficient to counteract the pro-inflammatory environment characteristic of psoriasis, underscoring the limited effectiveness of IL-10′s immunoregulatory function in this context. Treatment status was recorded in the study questionnaires, allowing for the consideration of its potential influence on cytokine levels.

Interestingly, no association was observed between IL-10 or TGF-β levels and the rs2280883 or rs3761548 variants of the *FOXP3* gene. This lack of correlation suggests that the influence of these variants on psoriasis risk may not involve the direct modulation of these cytokines at the systemic level. Instead, their effects may be more localized, potentially impacting *FOXP3* expression and regulatory T-cell (Treg) function within the skin or other immune-related tissues.

This study has several limitations. The wide confidence intervals in the odds ratios indicate variability, likely due to the small sample size, emphasizing the need for larger, more extensive studies. Additionally, the functional impact of the *FOXP3* variants, such as their influence on mRNA or protein expression, was not assessed, limiting mechanistic insights into how these variants may contribute to the pathogenesis of psoriasis. Moreover, cytokine measurements were performed at the systemic level and may not fully reflect local immune activity within psoriatic lesions. Furthermore, the use of drugs such as methotrexate among participants, which can modulate cytokine levels, was not controlled in this study. Although there were no significant differences in cytokine levels between the pharmacologically treated and untreated groups, this could still affect the interpretation of cytokine-related findings, representing a significant limitation.

Future research should prioritize investigating the localized effects of *FOXP3* variants on Treg function within psoriatic lesions, as well as their interaction with other key immunoregulatory molecules. Additionally, further exploration of the compensatory mechanisms of IL-10 in psoriasis could provide deeper insights into the interplay between systemic and local immune regulation in the disease.

## 4. Materials and Methods

### 4.1. Study Population

This study was conducted in accordance with the ethical guidelines of the Declaration of Helsinki (2024 revision) and was approved by the Ethics Committee of the University of Guadalajara (Approval No. CI-08918). Written informed consent was obtained from all participants prior to enrollment, as documented in Supplementary Material S1.

To ensure participant confidentiality, all personal data were anonymized, and information was securely managed in compliance with ethical and privacy regulations. This study posed a minimal risk as it involved only routine clinical evaluations and genetic analysis, with no invasive procedures beyond standard sample collection. Furthermore, to mitigate potential ethical concerns, the study was strictly limited to adult participants who were fully capable of providing informed consent, thereby ensuring that no vulnerable populations were involved.

The study included 101 PP patients and 106 control subjects (CSs). Patients were recruited from the Jalisco Dermatological Institute, “Dr. José Barba Rubio,” of the Mexican Ministry of Health in Guadalajara, Jalisco, Mexico, and all met the clinical and histopathological criteria for PP diagnosis. The CS group comprised self-reported healthy individuals from the general population, with no history of acute or chronic inflammatory or autoimmune conditions at the time of recruitment. Both the PP patients and CSs were unrelated individuals from the same Mexican population, all over 18 years of age. PP patients were exclusively diagnosed with psoriasis and had no other concomitant chronic or autoimmune diseases. To minimize population heterogeneity, only Mestizo individuals from Western Mexico were included in either of the study groups.

### 4.2. FOXP3 Variants Genotyping

Genomic DNA (gDNA) was extracted from peripheral blood leukocytes using Miller’s modified salting-out technique [43]. Blood samples were lysed with an equal volume of TTS buffer (Tris base, Triton X-100, and sucrose; Sigma-Aldrich [St. Louis, MO, USA]), homogenized by inversion, and centrifuged at 3000 rpm for 10 min. The leukocyte pellet was washed with TTS buffer, transferred to a 1.5 mL sterile tube, and centrifuged at 12,000 rpm for 2 min to remove erythrocytes. The pellet was then resuspended in 570 μL of 5 mM NaCl (Sigma-Aldrich; St. Louis, MO, USA), vortexed, and treated with 30 μL of 10% sodium dodecyl sulfate (Sigma-Aldrich; St. Louis, MO, USA) for cell lysis. Following homogenization, 200 μL of saturated NaCl was added and mixed for 15 min, followed by centrifugation at 11,500 rpm for 25 min at 4 °C. The supernatant was transferred to a 15 mL conical tube, where DNA precipitation was carried out using 2 mL of cold absolute ethanol (Sigma-Aldrich; St. Louis, MO, USA), followed by overnight incubation at −20 °C. The DNA pellet was washed 2–3 times with 70% cold ethanol, centrifuged at 9000 rpm for 10 min, and air-dried to remove residual ethanol. Finally, the DNA was resuspended in 200 μL of sterile water, and its concentration and purity were assessed by spectrophotometry at 260/280 nm.

The −3279 C>A (rs3761548) and IVS9+459 G>A (rs2280883) variants in the *FOXP3* gene were analyzed using polymerase chain reaction–restriction fragment length polymorphism (PCR-RFLP). The primers used for the amplification of both variants are listed in Table 3. These oligonucleotides were synthesized by T4 Oligo (Guanajuato, Mexico). The PCR conditions used for the amplification of both *FOXP3* variants are detailed in Table 4. All reagents were sourced from Invitrogen™ Life Technologies (Carlsbad, CA, USA).

**Figure 4:**
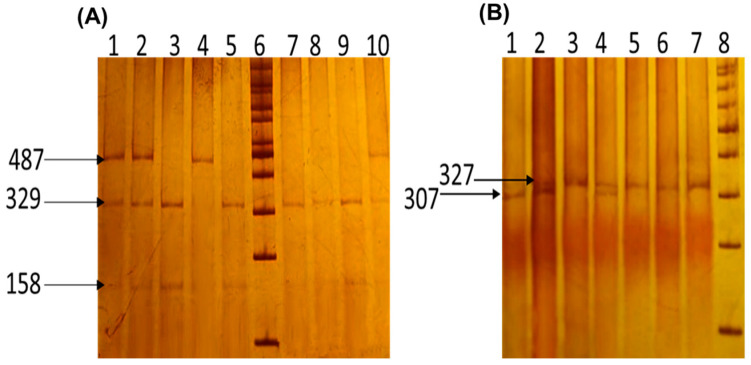
Genotypic identification of the *FOXP3* rs3761548 and rs2280883 variants. The images display polyacrylamide gels. (**A**) rs3761548: Lanes 1, 2, and 10 represent CA genotypes; lanes 3, 5, and 7–9 correspond to CC genotypes; lane 4 represents the AA genotype; lane 6 contains the 100 bp molecular weight marker. (**B**) rs2280883: Lane 1 represents the AA genotype; lanes 2 and 4 correspond to GA genotypes; lanes 3 and 5–7 represent GG genotypes; lane 8 contains the 100 bp molecular weight marker. The 20 bp band was not visible in the gel as it migrated beyond the detection range.

For the rs3761548 variant, the 487 bp fragment was digested using the *PstI* enzyme with NEBuffer 3.1 (New England BioLabs, Inc., Ipswich, MA, USA) and incubated at 37 °C for 6 h. The digested products were then separated by electrophoresis on a 6% polyacrylamide gel (29:1 acrylamide/bisacrylamide ratio; Sigma-Aldrich [St. Louis, MO, USA]) and stained with 2% AgNO_3_ (Sigma-Aldrich; St. Louis, MO, USA). The A allele was identified by a single 487 bp fragment, while the C allele was represented by 329 bp and 158 bp fragments (Figure 4A).

For the rs2280883 variant, the 327 bp fragment was digested using the *BsmI* enzyme with CutSmart buffer (New England BioLabs, Inc., Ipswich, MA, USA) following a 16 h incubation at 65 °C. The digested products were then separated by electrophoresis on a 6% polyacrylamide gel (29:1 acrylamide/bisacrylamide ratio) and stained with 2% AgNO_3_. The three possible genotypes were identified based on distinct band patterns observed on the gel: GG (327 bp), GA (327 bp, 307 bp, and 20 bp), and AA (307 bp and 20 bp) (Figure 4B).

Both the *BsmI* and *PstI* enzymes used in this study do not exhibit star activity, ensuring specificity in digestion. The selected buffer conditions allowed for overnight digestion without inducing star activity or causing DNA degradation. The reaction conditions were optimized to prevent nonspecific cleavage and to ensure accurate and reliable genotyping results. To verify the repeatability of the results, 5% of the samples from both the patient and control groups were genotyped twice, achieving 100% reproducibility.

### 4.3. Quantification of IL-10 and TGF-β Serum Levels

IL-10 and TGF-β serum levels were quantified using commercial ELISA kits: the Human IL-10 Quantikine HS ELISA Kit (Cat. HS100C; R&D Systems Inc., Minneapolis, MN, USA) and the Human TGF-β1 ELISA Kit (Cat. ab100647; Abcam, Cambridge, UK). The procedures were performed according to the manufacturer’s instructions. The assay sensitivities were 0.17 pg/mL for IL-10 and 18 pg/mL for TGF-β1.

### 4.4. Statistical Analysis

The genotypic and allelic frequencies of the variants were determined through direct counting. The Hardy–Weinberg equilibrium was assessed in the control group. Chi-square tests using 2 × 2 contingency tables were applied to analyze genotypic and allelic distributions. The association between variants and disease susceptibility was evaluated by calculating odds ratios (OR) with 95% confidence intervals, considering a significance threshold of *p* < 0.05. Haplotype frequencies and linkage disequilibrium (LD) were calculated using SHEsisPlus software [45]. All statistical analyses were performed using Stata 9.0 and GraphPad Prism 8.0.2.

## 5. Conclusions

A significant association was identified between the rs2280883 AA genotype of the *FOXP3* gene and an increased risk of PP in the Mexican population, whereas no such association was observed for rs3761548. Additionally, no linkage disequilibrium (LD) was detected between these variants, suggesting independent effects. Elevated IL-10 levels in PP patients likely reflect a compensatory response to chronic inflammation; however, these levels may be insufficient to counteract the disease’s pro-inflammatory environment. Notably, neither IL-10 nor TGF-β1 levels were associated with *FOXP3* variants, suggesting their influence on Treg function may be localized rather than mediated through systemic cytokine modulation. These findings underscore the importance of population-specific studies in elucidating psoriasis pathogenesis and genetic susceptibility.

## Figures and Tables

**Table 1:** Clinical and demographic characteristics of PP patients and CSs.

Characteristics	PP (n = 101)	CS (n = 106)	*p*-Value
Age ^a^	46 (35–57)	45 (32–51)	0.11
Gender, n (%)			
Female	49 (49)	60 (57)	0.24
Male	52 (51)	46 (43)	
BMI (kg/m^2^) ^b^	29.5 ± 4.8	28.7 ± 5.2	0.53
Time of evolution of the disease (years) ^b^	9 ± 0.98		
PASI^b^	10.9 ± 8.5		
Mild (<10), n (%)	40 (40)		
Moderate to severe (≥10), n (%)	61 (60)		
Treatment, n (%)			
Topical exfoliants (30% Urea + Dimeticone)	34 (33)		
MTX	11 (11)		
Topical steroids	5 (5)		
Topical exfoliants + MTX	8 (8)		
MTX + Topical steroids	5 (5)		
Topical exfoliants + MTX + Topical steroids	5 (5)		
None	33 (33)		

^a^ Data are presented as median (25th–75th percentile). ^b^ Data are expressed as mean ± standard error. *p*-values were calculated using the χ^2^ test or Wilcoxon–Mann–Whitney test, as appropriate. Abbreviations: CS, control subject; PP, plaque psoriasis; BMI, body mass index; PASI, Psoriasis Area and Severity Index; MTX, methotrexate.

**Table 2:** Genotype and allele frequencies of rs2280883 and rs3761548 *FOXP3* variants in PP patients and CSs.

Variants	PP n = 101% (n)	CS n = 106% (n)	OR (CI 95%)	*p*-Value
rs2280883				
Genotypes				
GG ^a^	56 (57)	63 (67)	1	-
GA	23 (23)	36 (38)	0.71 (0.38–1.33)	0.28
AA	21 (21)	1 (1)	24.68 (3.22–189.25)	**<0.0001**
Alleles				
G ^a^	68 (137)	81 (172)	1	-
A	32 (65)	19 (40)	2.04 (1.29–3.21)	**0.001**
Genetic models				
Dominant				
GG ^a^	56 (57)	63 (67)	1	-
GA+AA	44 (44)	37 (39)	1.32 (0.76–2.31)	0.32
Recessive				
GG+GA ^a^	79 (80)	99 (105)	1	-
AA	21 (21)	1 (1)	27.56 (3.63–209.24)	**<0.0001**
rs3761548				
Genotypes				
CC ^a^	59 (60)	58 (61)	1	-
CA	30 (30)	34 (36)	0.85 (0.46–1.56)	0.60
AA	11 (11)	8 (9)	1.32 (0.49–3.54)	0.56
Alleles				
C ^a^	74 (150)	75 (158)	1	-
A	26 (52)	25 (54)	1.04 (0.66–1.63)	0.86
Genetic models				
Dominant				
CC ^a^	59 (60)	58 (61)	1	-
CA+AA	41 (41)	42 (45)	0.94 (0.53–1.65)	0.83
Recessive				
CC+CA ^a^	89 (90)	92 (97)	1	-
AA	11 (11)	8 (9)	1.40 (0.54–3.65)	0.48

^a^ Genotype or allele of reference. *p*-values were calculated using the Chi-square test. Bold values indicate statistical significance at the *p* < 0.05 level. Abbreviations: PP, plaque psoriasis; CS, control subject; OR, odds ratio; CI, confidence interval.

**Table 3:** Primer sequences used for the amplification of the −3279 C>A (rs3761548) and IVS9+459 G>A (rs2280883) variants in the *FOXP3* gene.

*FOXP3* Variant	Primers	Amplicon Size
rs3761548	5′GCCCTTGTCTACTCCACGCCTCT3′ (sense) 5′ CAGCCTTCGCCAATACAGAGCC 3′ (antisense)	487 bp [44]
rs2280883	5′ TACACCCCCAACTGGGCAGC 3′ (sense) 5′ TGGGGTTCGGTGTGGAGTGA 3′ (antisense) (mismatch is underlined)	327 bp [27]

**Table 4:** PCR conditions used for the amplification of the −3279 C>A (rs3761548) and IVS9+459 G>A (rs2280883) variants in the *FOXP3* gene.

Reagents	Final Concentration	PCR Amplification Conditions
rs3761548		
Buffer 10×	1×	Initial denaturing: 95 °C, 5 min 35 cycles: Denaturing: 30 s at 94 °C Annealing: 35 s at 58 °C Extension: 40 s at 72 °C, Final extension: 5 min at 72 °C
MgCl_2_	2.5 mM	
dNTPs	0.4 mM	
Primers sense and antisense	0.15 µM	
Taq DNA polymerase	1.15 U/µL	
DNA	100 ng	
Water	(up to 15 µL)	
rs2280883		
Buffer 10×	1×	Initial denaturing: 95 °C, 5 min 40 cycles: Denaturing: 30 s, 94 °C Annealing: 35 s, 60 °C Extension: 30 s, 72 °C, Final extension: 10 min, 72 °C
MgCl_2_	2.5 mM	
dNTPs	0.4 mM	
Primer sense and antisense	0.15 µM	
Taq DNA polymerase	1.15 U/µL	
DNA	100 ng	
Water	(up to 15 µL)	

## Data Availability

The data supporting the findings of this study are available from the corresponding author upon reasonable request.

## Supplementary Materials

The supporting information can be downloaded at: https://www.mdpi.com/article/10.3390/ijms26051789/s1.
