# Supplementary material for: Influence of FOXP3 rs2280883 and rs3761548 Variants on IL-10 and TGF-β1 Serum Levels and Plaque Psoriasis Risk in the Mexican Population
Source: Int J Mol Sci. 2025 Feb 20;26(5):1789. doi: 10.3390/ijms26051789 (PMC11898888; doi:10.3390/ijms26051789)
Supplement: Supplementary file 1 [file ijms-26-01789-s001.zip › ijms-3471635-supplementary.pdf]

Supplementary Material S1

Consentimiento Informado

TÍTULO DEL PROYECTO DE INVESTIGACIÓN:

Asociación de los polimorfismos -3279 A>C (rs3761548) y IVS9+459 A>G (rs2280883) de *FOXP3* con la presencia de células T reguladoras CD4+CD25+FOXP3+ y los niveles de IL-10 y TGF-β en pacientes con psoriasis en placas del occidente de México

Investigador responsable: Dra en C Anabell Alvarado Navarro

Clave del paciente: ____________________

Por favor lea atentamente este documento

Usted está invitado a participar en este proyecto de investigación por tener un diagnóstico de Psoriasis en placas y ser mayor de 18 años. Se solicita lea el siguiente material para que pueda entender su participación en este proyecto y decida participar o no, de manera libre e informada. Por favor, haga cualquier pregunta que pueda tener al respecto.

Objetivo del estudio
Aunque no se conoce la causa de la psoriasis, se cree que los factores genéticos y ambientales favorecen la severidad de la enfermedad. Su participación en este proyecto ayudará a comprender si la psoriasis pudiera estar relacionada con condiciones hereditarias.

¿En qué consiste su participación?

- Se le realizará un examen físico realizado por un médico dermatólogo y se tomarán mediciones incluyendo: peso, talla, circunferencia de cintura, cadera y tensión arterial, con el fin de conocer su estado general de salud.
- Se le extraerá una muestra de sangre: Se usará una aguja para extraer sangre de una vena del brazo. Se extraen un total de aproximadamente de 20 mL de sangre (equivale a 1 cucharada y media aproximadamente) durante todo el estudio. En ocasiones, puede ser necesario repetir una toma de sangre del mismo volumen en caso de degradación de la misma.
- Es necesario estar en ayuno antes de la visita: ésto significa que durante 8 horas no ingerirá ningún alimento o bebida excepto agua, o sea que usted debe ayunar durante la noche antes de la toma de sangre.
- Sus muestras de sangre se usarán para la investigación científica/genética. Pueden analizarse distintas sustancias en sus muestras.
- La duración de su evaluación, interrogatorio y toma muestra no será mayor a 30 minutos.
  La investigación genética es el estudio del ADN. El ADN porta la información que determina nuestros rasgos. Por ejemplo, nuestro ADN determina el color de nuestro cabello y nuestros ojos. El ADN también puede explicar por qué algunas personas responden a algunos medicamentos y otras no. También puede explicar por qué algunas personas desarrollan ciertas enfermedades y otras no.

¿Existen posibles riesgos e inconvenientes para usted?
Usted pudiera presentar en el sitio del piquete de aguja cierto tipo de molestias locales, ligero enrojecimiento de la zona y en el menor de los casos una pequeña mancha tipo moretón que no durará más de 5 días. De presentarse estos, contará con la atención necesaria del personal donde se tomen las muestras. El piquete no representa ningún daño a su salud ni impedimento a su vida cotidiana.

Además:
- Este estudio no exige que su médico cambie el tratamiento que usted recibe para su cuidado regular.
- Cualquier cambio en su tratamiento será una decisión tomada por su médico y no estará relacionada a su participación en este estudio.

¿Recibe usted algún beneficio por participar?
Usted seguirá recibiendo la atención médica que requiera por su Médico tratante.
Usted no pagará por su participación en este estudio. Además, es posible que los resultados de este estudio puedan ayudar a los pacientes en el futuro.
Usted no recibirá un pago por participar en este estudio.
Los investigadores atenderán cualquier pregunta o aclaración acerca del proyecto.
Su participación no obliga a los responsables del proyecto a otorgarle atención a los problemas de salud diagnosticados a partir de la toma de la muestra. Sin embargo, en caso de presentar alguna alteración, se le derivará con su médico tratante.
Usted no recibirá los resultados obtenidos a partir del análisis de su muestra, a menos que los solicite por escrito.

¿Sus datos se mantendrán confidenciales?
- La información se almacenará tanto en papel como en una computadora. Para proteger su privacidad, la información se guardará de forma que no se le pueda identificar. Si se publican los resultados del estudio, su identidad será confidencial. Al firmar este formulario, autoriza el uso de su información.
- El Médico a cargo del estudio guardará su historia clínica y una lista que relaciona el nombre de cada paciente con su código numérico durante 5 años.
- Los miembros del Comité de Ética así como los empleados del lugar donde se lleva a cabo el estudio y los investigadores tendrán acceso a la lista y podrán comparar y revisar la información reunida sobre usted utilizando su historia clínica. Su historia clínica no se hará pública.
- Para proteger su privacidad, sus muestras serán etiquetadas con su número del estudio. Los científicos que realizan la investigación no conocerán su identidad.
- Al firmar este formulario, permite que las personas que tengan una razón legítima puedan tener acceso directo a su historia clínica.
- Los resultados del estudio se le proporcionarán a usted o a su médico tratante, en caso de ser solicitado.
- Los resultados de las pruebas realizadas en sus muestras son sólo para investigación. No influirán para su atención médica. No se usarán para hacer un diagnóstico de su estado de salud.

¿Cómo se conservarán y manejarán sus muestras?
- Todas sus muestras podrían conservarse y usarse hasta el término de este estudio. Esto permitirá realizar esta investigación. El investigador garantizará que sus muestras se conserven

congeladas. Sus muestras serán destruidas al concluir el estudio (aproximadamente un año). No se le informará cuando se destruyan sus muestras. Sin embargo, se mantendrá informado anualmente al Comité de ética del Instituto Dermatológico de Jalisco.

- Usted puede retirar su consentimiento para el uso de sus muestras en cualquier momento y por cualquier razón, en tal caso, sus muestras serán desechadas incluso antes de ser evaluadas. Esto no afectará su acceso a la atención médica y al cuidado que recibe. Para retirar su consentimiento, lo hará por escrito y dirigido a la Dra. Miriam Sarahí Preciado Aguiar o con la Dra. Anabell Alvarado Navarro, responsables del proyecto.
- Sus muestras no serán vendidas, prestadas o entregadas a ningún grupo independiente para su uso. Los otros investigadores que trabajan con el investigador responsable de este estudio, no están autorizados para compartir las muestras con ningún tercero que no esté autorizado. El investigador responsable controlará lo que se haga con sus muestras, con el resguardo institucional al que pertenecen los pacientes.
- Las muestras serán utilizadas exclusivamente con fines de investigación científica orientada a la salud. **Bajo ninguna circunstancia** serán usadas con fines comerciales.

¿Si decide no seguir participando?

- Su participación en este estudio es voluntaria. Puede aceptar participar en el estudio ahora y cambiar de opinión después. Usted puede suspender su participación en cualquier momento. Su decisión no afectará su cuidado regular. No afectará el que usted obtenga todo el cuidado que debe recibir.

Si otorga su consentimiento, por favor lea y luego firme abajo

Esta forma de consentimiento contiene información importante. Le ayudará a decidir si quiere participar en este estudio. Si todavía tiene alguna pregunta, por favor consulte al Médico del estudio o a uno de los miembros del personal del estudio antes de firmar esta forma.

Acuerdo para participar en el estudio:

- He leído esta información.
- Está redactada con un lenguaje que puedo leer y entender.
- Se me ha explicado este estudio.
- Se han contestado a mi satisfacción todas mis preguntas acerca del estudio y los posibles riesgos.
- Con base en esta información, acepto voluntariamente participar en este estudio.

Nombre y dirección de la persona otorgante del consentimiento	Firma
Nombre y dirección del testigo:	Firma
Nombre y dirección del testigo:	Firma

Al firmar esta forma, no he renunciado a ninguno de mis derechos legales que de otro modo tendría como un participante en un estudio de investigación. Por lo tanto no tengo ningún inconveniente en contestar el cuestionario adicional que me presenten para el registro de mi participación.
También he recibido una copia de esta forma de consentimiento.

______________________________ ____________

Nombre del Investigador Responsable Firma Fecha

En caso de alguna duda sobre el estudio comunicarse con la Dra. en C. Anabell Alvarado Navarro Investigador responsable en el horario de 9:00 am a 2:00 pm, Laboratorio de Inmunología CIINDE (33)36722848. Instituto Dermatológico de Jalisco ¨Dr. José Barba Rubio¨ Av. Federalismo Norte 3102 Col. Atemajac del Valle, Zapopan Jalisco C.P 45190.
